# P-402. Current Practices for the Outpatient Diagnosis & Management of Bacterial Tracheitis (BT) in Ventilator-Dependent Children (VC)

**DOI:** 10.1093/ofid/ofaf695.619

**Published:** 2026-01-11

**Authors:** Genevieve Donahey, Glenn J Rapsinski, Jessica Reyes-Angel, Michael D Green, Hiren Muzumdar

**Affiliations:** Children's Hospital of Pittsburgh, Pittsburgh, PA; Children's Hospital of Pittsburgh of UPMC, Pittsburgh, Pennsylvania; Children's Hospital of Pittsburgh, Pittsburgh, PA; University of Pittsburgh School of Medicine, Pittsburgh, PA; Children's Hospital of Pittsburgh, Pittsburgh, PA

## Abstract

**Background:**

There are no nationally recognized guidelines for the diagnosis & management of ventilator-associated BT. Guidelines for pediatric inpatient ordering of endotracheal cultures (EC) have reduced unnecessary cultures, but there are no studies examining outpatient practices & management of BT. We propose to extrapolate this work to the outpatient setting for VC.

**Table 1.**
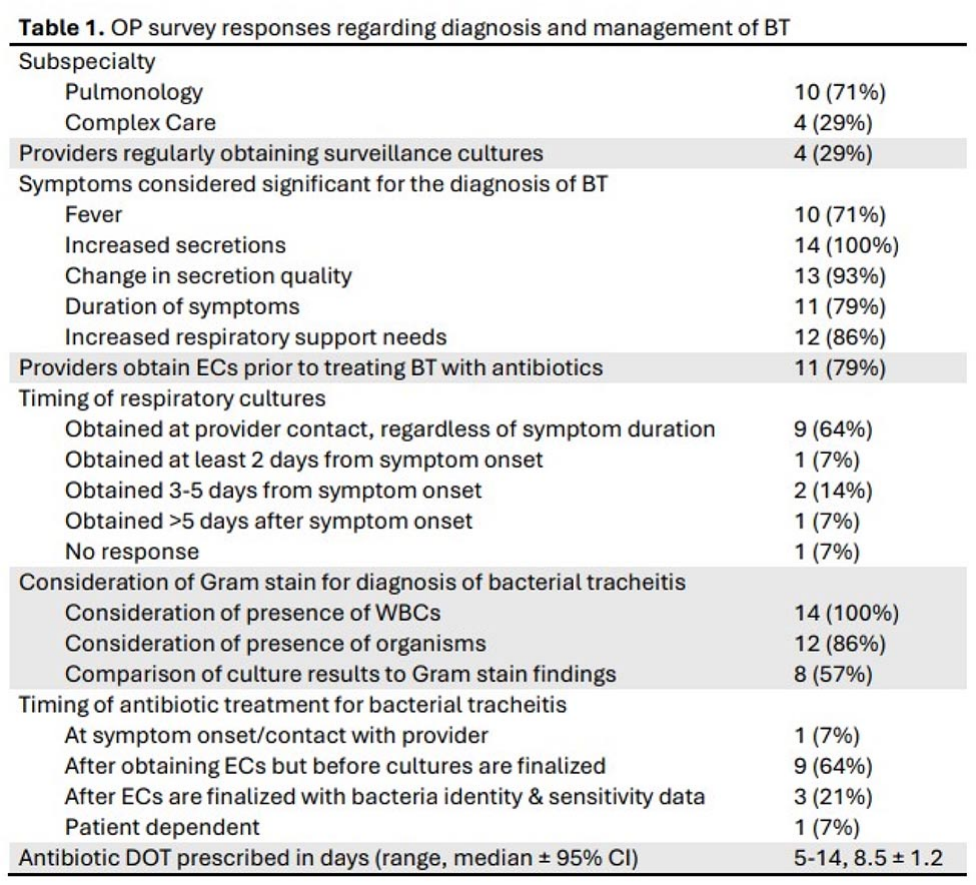
OP survey responses regarding diagnosis and management of BT.

**Table 2.**
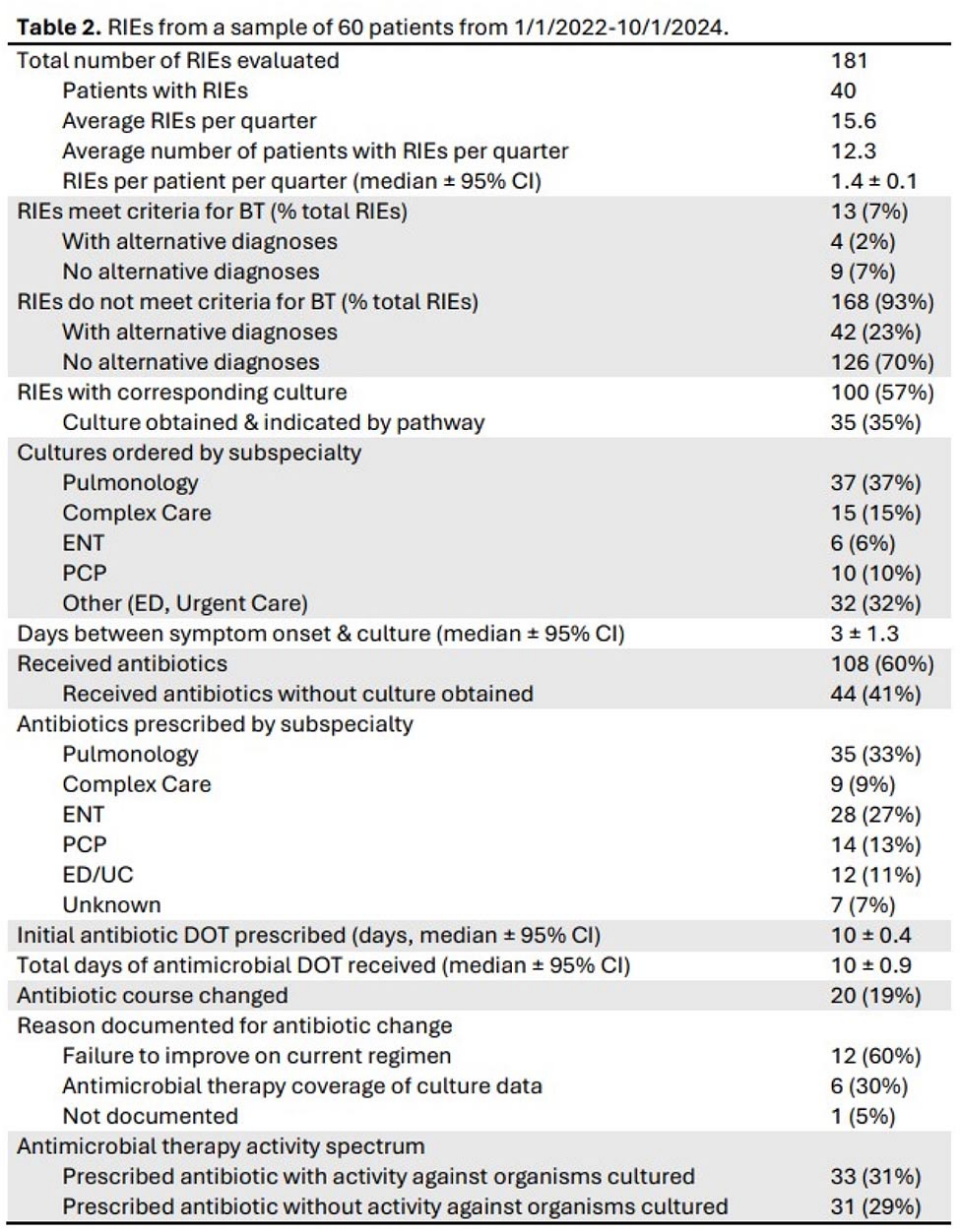
RIEs from a sample of 60 patients from 1/1/2022-10/1/2024.

**Methods:**

This project examines outpatient provider (OP) practices via self-reported survey responses & chart review of respiratory illness events (RIE) in VC. OP RIEs were characterized using standardized definitions. Medical records were reviewed from 1/1/2022-10/1/2024 to examine the number of & reason for ordering ECs, specific antibiotic treatment, both prescribed & actual days of therapy (DOT), & outcomes including ED visits & hospitalization.

**Results:**

18 OPs were identified in the pulmonology & complex care departments. Baseline practice surveys distributed with a 78% response rate. 11/14 of OPs report routinely obtaining ECs to assist with diagnosis and management of BT & 4/14 obtain routine surveillance cultures (Table 1). Antibiotic DOT had a large range, 5-14 days (Table 1). Survey responses were compared with data obtained from 145 VC, including 220 ECs obtained from 1/1/2022-10/1/2024, & 174 RIEs from a subset of 60 VC (Table 2). 100 ECs were associated with RIEs. 33/220 were surveillance cultures, ordered by Pulmonology only. 9 cultures had no indication documented. The highest proportion of cultures ordered in assessment of RIEs were ordered by Pulmonology & the ED, while ENT had the lowest. Pulmonology & ENT had the highest rates of antibiotic prescriptions.

**Conclusion:**

Survey data and chart review indicates that there is significant practice variation regarding diagnosis & treatment of bacterial tracheitis & the use of ECs. These results reflect the known lack of diagnostic & treatment data regarding BT in VC, thus presenting an opportunity for meaningful intervention to standardize care.

**Disclosures:**

Michael D. Green, MD, MPH, ADMA: Advisor/Consultant|Bristol Myers Squibb: Advisor/Consultant|ITB-MED: Advisor/Consultant

